# Dominated Spider Species and the Predation Assessment on *Apolygus lucorum* (Hemiptera: Miridae) in a Tea Plantation

**DOI:** 10.1002/ece3.73078

**Published:** 2026-02-10

**Authors:** Meng Zhang, Xiaodong Fu, Feiyang Li, Yalan Zhu, Yu Gao, Dayu Zhang, Qian Wang

**Affiliations:** ^1^ Zhejiang Key Laboratory of Biology and Ecological Regulation of Crop Pathogens and Insect, College of Advanced Agricultural Sciences Zhejiang A&F University Hangzhou China; ^2^ Agricultural and Rural Bureau of Lanshan District Rizhao China; ^3^ State Key Laboratory for Biology of Plant Diseases and Insect Pests, Institute of Plant Protection Chinese Academy of Agricultural Sciences Beijing China

**Keywords:** *Apolygus lucorum*, functional responses, predation, spiders, tea

## Abstract

The mirid bug *Apolygus lucorum* (Meyer‐Dür), a major pest affecting tea, also poses significant threats to a wide range of other crops across China. Identifying the dominant predatory spiders of the mirid bug and their pest control functions can provide a scientific basis for developing biological control technologies for this pest in tea plantations. In this study, we calculated the dominant presence of these spider species and evaluated the phenological overlap with the population of *A. lucorum* . Additionally, DNA from field‐collected specimens of spider species was analyzed by *A. lucorum* ‐specific primers to detect the presence of residual DNA from the mirid bug. Using the predator–prey functional response model, the predation efficiency of various adult spider species on *A. lucorum* was assessed in laboratory conditions by testing different prey densities. The results showed that the greatest temporal niche overlap was observed between *Xysticus ephippiatus* and *A. lucorum.* Molecular detection results showed that *X. ephippiatus* and *Misumenops tricuspidatus* had significantly higher detection rates of the mirid bug than other spiders. The functional response of lab predation indicates that the predation ability of different spider species on *A. lucorum* increases with the density of prey. The predation functions of both adult *X. ephippiatus* and *M. tricuspidatus* conform to the Holling‐II model. At a high prey density, the predation quantity of adult *X. ephippiatus* is significantly higher than that of adult *M. tricuspidatus*. In conclusion, the spider *X. ephippiatus* demonstrates the greatest potential as a biological control agent against *A. lucorum* within an integrated pest management framework. This research offers valuable scientific insights for leveraging predator species to effectively manage *A. lucorum* populations in tea plantations.

## Graphical abstract

The mirid bug *Apolygus lucorum* (Meyer‐Dür) is a major pest affecting tea. Identifying the dominant predatory spiders of the mirid bug can provide a scientific basis for developing biological control technologies. *Xysticus ephippiatus* demonstrates the greatest potential as a biological control agent against *A. lucorum.*

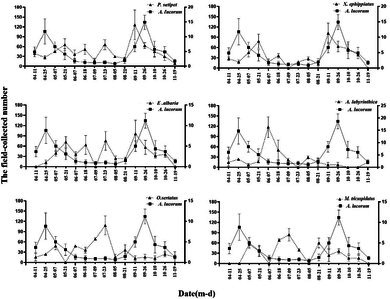

## 1 Introduction

The tea plant ( *Camellia sinensis* (L.) O. Kuntze) is a globally cultivated, leaf‐based woody crop of significant economic importance (Ali 1970; Weisburger 1997). The quality and yield of tea are negatively impacted by several pest species (Hazarika et al. 2009). At present, pesticides are the most commonly applied to defend the tea crop against the tea pests (Hazarika et al. 2009; Settele et al. 2008), however, this application has the potential to cause environmental contamination, pesticide resistance, damage to the nontarget beneficial arthropods, and undesirable residues on made tea (Chaudhuri 1999; Cranham 1966; Sivapalan 1999). At the same time, increased tea consumption necessitates higher standards for tea pest management.

Biological control is an ecological service provided by various organisms, such as arthropods, that plays a significant role in managing crop pests (Jonsson et al. 2008; Wyckhuys et al. 2013). Before implementing predator‐based pest control strategies, it is essential to conduct field‐based identification of the primary predatory species targeting the pest population. But it is difficult to accurately identify arthropod predation under field conditions, as predators and their prey generally exhibit small body sizes, possess high mobility, and demonstrate nocturnal behavioral patterns (Greenstone and Morgan 1989; Hoogendoorn and Heimpel 2001; Li et al. 2017). A frequently employed approach to evaluating predation involves dissecting the guts of predators to identify prey remnants. Nevertheless, this technique is ineffective for piercing‐sucking predators, as they ingest bodily fluids rather than solid prey particles, leaving no distinct prey remains in their guts (Cohen 1995; Furlong 2015). As a highly efficient alternative, the analysis of gut DNA content allows for the accurate identification of minute traces of target DNA from the diets of pests, establishing it as the most widely employed approach for investigating predator–prey relationships (Greenstone et al. 2014; Li et al. 2017; Symondson et al. 2002).

The green mirid bug, *Apolygus lucorum*, is an omnivorous agricultural pest in China, which has resulted in extensive yield losses among more than 200 species of plants, including cotton, fruit, vegetables, and many other cash crops (Lu et al. 2010). In recent years, the mirid bug has become a significant piercing‐sucking pest impacting tea plants (Lun et al. 2024). It damages tea plants by piercing and sucking young buds and tender leaves. The affected buds and leaves exhibit slow growth and poor ability to retain tenderness, leading to processed dry tea that is brittle in appearance, with a bland and astringent taste, resulting in the destruction of 30%–40% of young tea shoots throughout the spring growth period (Tian et al. 2019; Zhang et al. 2017). As the period of damage by overwintering nymphs to the tea buds is highly consistent with the spring tea picking time, the use of chemical pesticides for control is limited due to pesticide residues. Meanwhile, it is difficult to control due to its extraordinary mobility and ability to hide. The development and implementation of effective, environmentally sustainable approaches for managing *A. lucorum* are critically needed to enhance the quality and safety of tea production.

In Chinese tea plantations, more than 1100 species of natural enemies have been documented, among which arthropod predators are the dominant group, making up 54.5% of the total (Yang et al. 2017a; Ye et al. 2014). Among these predators, spiders are the primary predatory natural enemies. Numerous field studies examining the population dynamics between spiders and pests have demonstrated that spiders significantly contribute to biological control efforts (Marc et al. 1999; Nyffeler and Benz 1987; Wen et al. 2009). However, field evidence is often insufficient to confirm whether, and to what extent, spiders prey on the targeted pest species. Therefore, thoroughly examining the actual predator–prey interactions between spiders and pests, along with measuring predation efficiency, is essential for gaining a deeper understanding of the potential contributions spiders can make to biological pest control (Greenstone 1999; Yang et al. 2017b).

In this study, we systematically investigated the population dynamics of *A. lucorum* and its major predatory spiders in tea plantations. Molecular detection was performed on spider samples collected from the tea gardens to construct the predator–prey relationship between the predatory spiders and the green mirid bug. Laboratory experiments were conducted to evaluate the predation capabilities of different spider species on the green mirid bug through functional response analysis. This study systematically elucidates the control effects of various natural enemies on the green mirid bug, establishing a scientific framework for the progressive development of eco‐friendly pest management solutions in the tea industry.

## 2 Materials and Methods

### 2.1 Insects

The mirid bug *A. lucorum* colonies were established at Zhejiang A & F University (119.7° E, 30.2° N). The mirid bugs were maintained in plastic containers measuring 20 × 10 × 6 cm. Green bean pods ( *Phaseolus vulgaris* L.) served as the primary food source for the mirid bugs, supplemented for adults with cotton balls dipped in a 10% sucrose solution. These containers, housing the *A. lucorum* colonies, were kept in growth chambers under regulated conditions: a temperature of 29°C ± 1°C, 65% ± 5% relative humidity, and a light–dark cycle of 14 h of illumination followed by 10 h of darkness.

The specificity of the primers was evaluated using non‐target arthropod species collected from tea plantations located at Zhejiang A&F University, situated in Hangzhou City, Zhejiang Province, China (119.7° E, 30.2° N). These arthropods were individually placed in 2 mL centrifuge tubes and deprived of food for 2 or 7 days to eliminate any residual prey DNA from their guts. Following this, they were frozen and stored at −20°C until DNA extraction was performed.

### 2.2 Field Sampling

The research was carried out in a tea plantation in Yuhang, Hangzhou City, Zhejiang Province, China (119.7° E, 30.3° N), where conventional agronomic methods were followed, and insecticides were avoided throughout the sampling phase. Spiders and *A. lucorum* were sampled biweekly from April to November 2021. Three rows, each spaced more than 10 m apart, were chosen, and within each row, 30 sub‐plots (measuring 2 × 2 m^2^) were randomly selected, with a minimum spacing of 1 m between them. Spiders and *A. lucorum* were sampled using a 38 cm diameter sweep net across the upper layer of tea canopies. The collected spiders and *A. lucorum* were then counted. Subsequently, the spiders were screened and used for subsequent DNA detection analysis. Each collected sample was individually transferred to 2 mL microcentrifuge tubes. Before initiating DNA extraction procedures, each predator sample underwent two successive washes with double‐distilled water (ddH_2_O) to ensure surface contamination removal. The samples were individually placed in 2 mL microcentrifuge tubes filled with 95% ethanol and maintained at −80°C for preservation before DNA extraction.

### 2.3 DNA Extraction

The DNA of *A. lucorum* and other arthropod species was extracted from whole bodies. DNA was extracted by a FastPure Blood/Cell/Tissue/Bacteria DNA isolation Mini Kit (Nanjing Vazyme Biotech Co. Ltd., Nanjing, China), following the protocol provided by the manufacturer. To ensure the integrity of the DNA extraction process, two negative control samples were included, using PCR‐grade water instead of DNA extract to verify that no contamination was present.

### 2.4 Primer Design

The COI gene was selected as the molecular barcode for species identification of *A. lucorum* , with the corresponding gene sequences being retrieved from GenBank (KC894257). Using BioEdit sequence alignment editor 7.1.3.0 (Hall 1999), the COI genes of *A. lucorum* and non‐target arthropods were aligned, and Primer Premier 5 version 5.00 (Lalitha 2000) was employed to design the specific primers listed in Table 1.

**TABLE 1:** Primer sequences and amplicon size of *A. lucorum* .

Primer name	Sequence (5′‐3′)	Annealing T (°C)	Size (bp)
*Aluc‐COIF*	CACTCTCTGCAAATATCTCA	59	191
*Athe‐COIR*	CAATAGGGCAGTAATTCCT		

### 2.5 PCR and Electrophoresis

Following the extraction of genomic DNA from field‐collected predators, the presence of *A. lucorum* DNA within the guts of spiders was determined through a species‐specific PCR assay designed for *A. lucorum* detection. To verify the success of the PCR amplification, two positive *A. lucorum* DNA samples were included in each assay. Additionally, two negative controls, where PCR‐grade water was substituted for the insect DNA extract, were incorporated to confirm the absence of any DNA contamination.

The PCR reaction was conducted in a total volume of 20 μL, containing the following components: 1 μL of DNA extract, 2 μL of 10 × Taq buffer (supplied by TransGen Biotech, Beijing, China), 0.4 μL of dNTP mix, 0.2 μL of Easy Taq DNA polymerase (TransGen Biotech, Beijing, China), 0.75 μL each of forward and reverse primers (at a concentration of 10 μM), and 14.9 μL of sterile distilled water to bring the total volume to 20 μL. The PCR reactions were performed in a Veriti 96‐Well Thermal Cycler (Applied Biosystems, United States). The amplification protocol began with an initial denaturation at 95°C for 10 min, followed by 35 cycles of denaturation at 95°C for 30 s, annealing at 58°C for 30 s, and extension at 72°C for 1 min. The process concluded with a final extension step at 72°C for 10 min. Following amplification, 6 μL of the PCR products were separated on a 2% agarose gel and visualized under a UV transilluminator.

### 2.6 Prey DNA Detectability Half‐Life Determination

To investigate whether the DNA of *A. lucorum* could be identified in the gut of its predators, the feeding experiments were conducted by the six dominant spider adults (*Plexippus setipes* (Karsch), *Xysticus ephippiatus* (Simon) (Thomisidae), *Agelena labyrinthica* (Clerck), *Evarcha albaria* (L. Koch), *Oxyopes sertatus* (L. Koch), and *Misumenops tricuspidatus*), which were collected from the tea plantation. Each spider was reared individually in a 2 × 10 cm glass tube, with a moistened sponge placed at the base to maintain optimal humidity levels. The feeding trial was conducted to assess the persistence of detectable *A. lucorum* DNA in spider specimens across different post‐ingestion time periods. Prior to the feeding experiments, all spiders were starved for 7 days at a controlled temperature of 25°C ± 1°C. Following the starvation period, each spider was supplied with ten second‐instar *A. lucorum* and permitted to feed for 1 h. Spiders that did not exhibit feeding behavior were excluded from the study. Post‐feeding, the detection efficiency was evaluated across six spider species at time intervals of 0, 12, 24, 36, 48, 60, and 72 h (10 individuals per time point). During the feeding process, the spiders were maintained in lab conditions at a temperature of 25°C ± 1°C, a relative humidity of 80%–85%, and a photoperiod of L12:D12 hours. Once each feeding interval was completed, the spiders were individually placed in 2 mL microcentrifuge tubes with 95% ethanol, kept at −80°C, and later processed for DNA extraction according to the described procedure. After extracting DNA from the spiders, *A. lucorum* DNA in their guts was analyzed via the specific PCR assay described in the previous section.

### 2.7 Functional Responses Study

A feeding experiment was conducted to assess the functional response of two generalist predatory spider species, *X. ephippiatus* and *M. tricuspidatus*, towards *A. lucorum* . Densities of 20, 30, 40, 50, and 60 s‐instar nymphs of *A. lucorum* per glass bottle (4 × 10 cm) were tested. A green bean pod (about 7 cm × 1 cm) with *A. lucorum* at different densities was placed in a glass bottle. Each spider was starved for 72 h and then placed in each glass bottle. The number of *A. lucorum* consumed was measured and documented after a 24‐h period. The study was conducted by repeating the experiment 10 times for each prey density. Prey were introduced into glass bottle (4 × 10 cm) at specified densities (20, 30, 40, 50, and 60 per bottle) in the absence of spider to establish control conditions, and the rates of natural mortality were then monitored and recorded.

### 2.8 Statistical Analyses

All statistical analyses were performed using SAS 9.2 statistical software (SAS Institute, Cary, NC, United States). The temporal niche overlap between spider species and *A. lucorum* was calculated using the approach introduced by (Hurlbert 1978):

$$L_{\mathrm{ij}}=S\Sigma_{h=1}^{s}P_{\mathrm{ih}}P_{\mathrm{jh}},$$

where *L*_*ij*_ is the Hurlbert niche overlap measure of species *i* on species *j*, *S* is the unit number of the resource sequence, and *P*_*ih*_ is the proportion of resource *h* to the total resource that species *i* utilizes and *P*_*jh*_ is the proportion of resource *h* to the total resource that species *j* uses. One‐way ANOVA was used to compare the digestion rates of different dominant spider species on *A. lucorum* . Using a previously published method (Chen et al. 2000; Gagnon et al. 2011), we weighted the detection rates to better assess the extent of DNA in the gut contents under natural conditions. Specifically, DNA with a shorter DS_50_ was given a weighting value of 1.0, and the weighting values for DNA from other spiders were derived by dividing this benchmark DS_50_ by the DS_50_ of the corresponding spider. A chi‐square test was conducted on the detection frequency of *A. lucorum* within six spider species. Based on the predation data of different spiders on *A. lucorum* obtained under laboratory conditions, the relationship between predation quantity and *A. lucorum* density was plotted. The Holling disk equation was then employed to analyze the functional response of predation. The Holling type II equation is Na=a′NT1+a′ThN, where Na represents the net rate of prey consumption by the predator over a specified time period, where a' denotes the instantaneous attack rate, N stands for prey density, T refers to the total predatory time of the predator (1 day), and Th indicates the time needed to handle and consume a mite at various developmental stages (handling time) (Hall 1999; Wang et al. 2019). A *t*‐test was used to compare the differences in predation rates of *A. lucorum* by different spider species at the same prey density.

## 3 Results

### 3.1 Specificity of *A. lucorum* Prey Primers

A specific primer set targeting the COI gene of *A. lucorum* (Table 1) was developed to facilitate the molecular identification of the mirid bug. The specificity of the PCR assay was verified by testing its ability to amplify the target gene segment in *A. lucorum* and 38 other non‐target arthropods (Table 2). The expected 191 bp COI gene fragment was amplified exclusively in *A. lucorum* , with no amplification detected in the 38 non‐target species or the negative controls. These findings indicate that the primers are specific to the species and can be effectively used for the accurate identification of *A. lucorum* at the molecular level.

**TABLE 2:** Invertebrate species used for the primers' specificity test.

Class	Order	Family	Species
Insecta	Hemiptera	Aphididae	*Toxoptera aurantii* (Boyer de Fonsco10mbe)
			*Aphis craccivora* Koch
		Cicadellidae	*Empoasca onukii* (Matsuda)
		Miridae	*Apolygus lucorμm* (Meyer‐Dür)
		Pentatomidae	*Halyomorpha halys* (Stål)
		Lygaeidae	*Geocoris pallidipenn*is (Costa)
		Aleyrodidae	*Aleurocanthus spiniferus* (Quaintance)
		Coccidae	*Ceroplastes japonicus* Green
		Aleyrodidae	*Aleurocanthus spiniferus* (Quaintanca)
	Thysanoptera	Thripidae	*Dendrothrips minowai* Priesner
	Coleoptera	Coccinellidae	*Harmonia axyridis* (Pallas)
			*Propylaea japonica* (Thunberg, 1781)
		Curculionidae	*Myllocerinus aurolineatus* Voss
	Lepidoptera	Geometridae	*Ectropis obliqua* Prout
			*Ectropis grisescen*s Warren
			*Scopula subpunctaria* (Herrich‐Schaeffer)
			*Jankowskia athlete* Oberthur
		Lymantriidae	*Euproctis pseudoconspersa* Strand
		Gracilariidae	*Caloptilia theivora* (Walsingham)
		Limacodidae	*Thosea sinensis* Walker
	Neuroptera	Chrysopidae	*Chrysoperla sinica* (Tjeder)
			*Chrysopa pallens* (Rambur)
	Diptera	Syrphidae	*Episyrphus balteatus* (De Geer)
			*Eupeodes corollae* Fabricius
Arachnida	Araneae	Salticidae	*Evarcha albaria* (L. Koch, 1878)
			*Plexippus setipet* Karsch, 1879
			*Phintella bifurcilinea* (Boes. et Str., 1906)
			*Carrhotus xanthogramma*
			*Phintella yinae*
		Globulidae	*Coleosoma octomaculatum*
		Araneidae	*Araneus ejusmodi* Boes. et Str., 1906
			*Neoscona theisi* (Walckenaer, 1842)
		Aracidae	*Misumenops tricuspidatus*
		Agelenidae	*Agelena labyrinthica* Clerck, 1758
		Clubionidae	*Clubiona reichlini* Schenkel, 1944
		Thomisidae	*Xysticus ephippiatus* (Simon)
		Oxyopidae	*Oxyopes sertatus* L. Koch, 1877
		Sioscardidae	*Tetragnatha maxillosa* (Thoren, 1895)
	Trombidiformes	Eriophyidae	*Acaphylla theae* Watt

### 3.2 DNA Detectability Half‐Life

**FIGURE 1:**
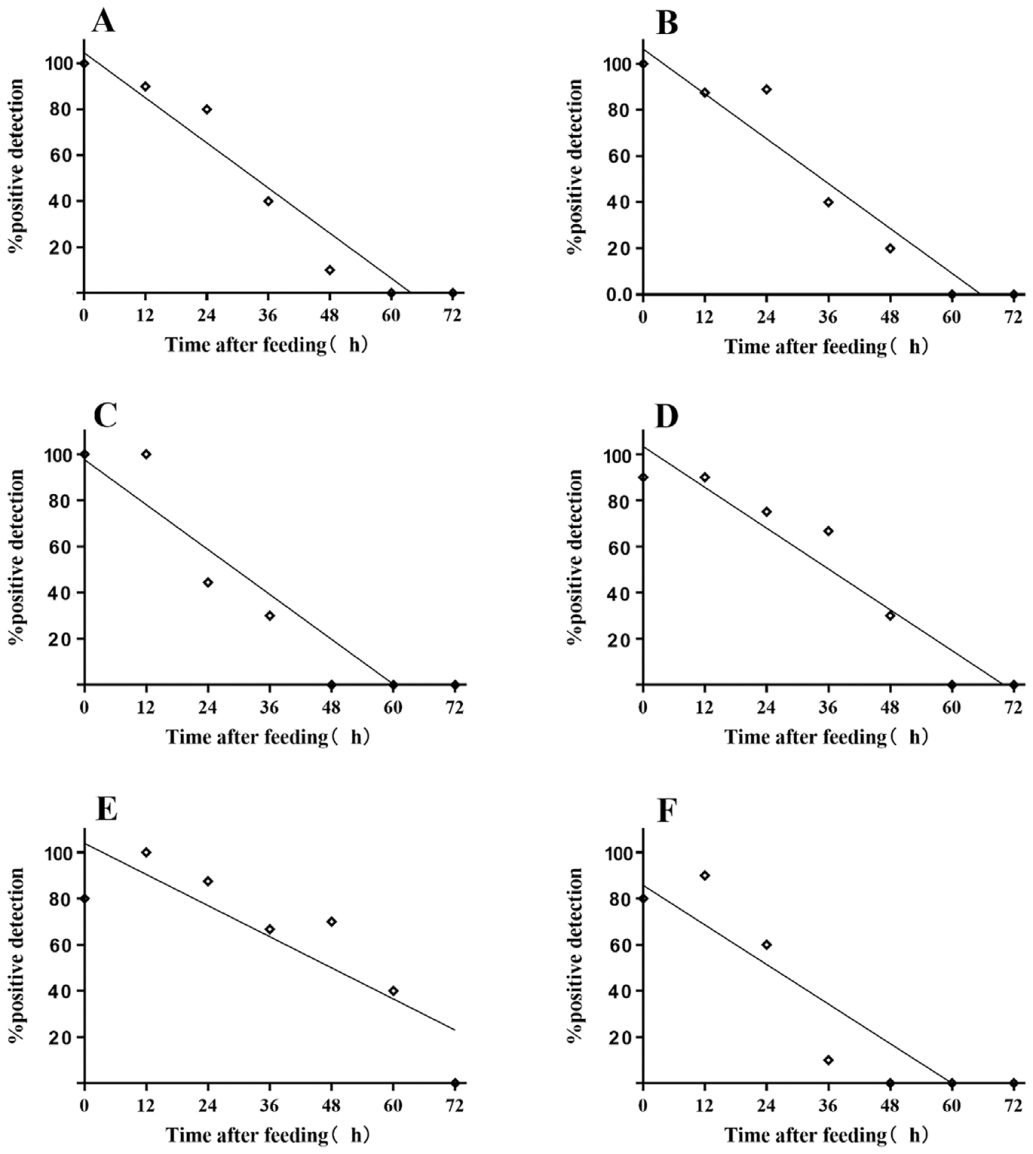
Detection of *A. lucorum* DNA in the six dominant spider species at different times after ingestion in a laboratory feeding trial.

The half‐life of prey DNA detectability (DS_50_) was assessed. As depicted in Figure 1, following feeding, the detectability of *A. lucorum* DNA progressively decreased as digestion time advanced (*X. ephippiatus Y* = 1.063–0.01619*X*; *R* ^2^ = 0.927; *M. tricuspidatus Y* = 1.039–0.01124*X*, *R* ^2^ = 0.747; *O. sertatus* *Y* = 0.857–0.014*X*, *R* ^2^ = 0.825; *A. labyrinthica* *Y* = 1.033–0.01473*X*, *R* ^2^ = 0.921; *P. setipes* *Y* = 1.046–0.01637*X*, *R* ^2^ = 0.933; *E. albaria* *Y* = 0.9754–0.01620*X*, *R* ^2^ = 0.873). Additionally, it reveals that the digestion rates of the six spider species for processing *A. lucorum* were not significantly different statistically (*F* = 2.38, *p* = 0.0669). The detectability half‐life of a second‐instar nymph of *A. lucorum* in the guts of *X. ephippiatus* , *M. tricuspidatus*, *O. sertatus* , *A. labyrinthica* , *P. setipes* , and *E. albaria* was determined by regression equations to be 34.77, 47.95, 25.5, 36.18, 33.35, and 29.35 h, respectively.

### 3.3 The Dominance and Population Dynamics of Spider Species, Along With Their Temporal Niche Overlap Values in Relation to *A. lucorum* in the Field

**FIGURE 2:**
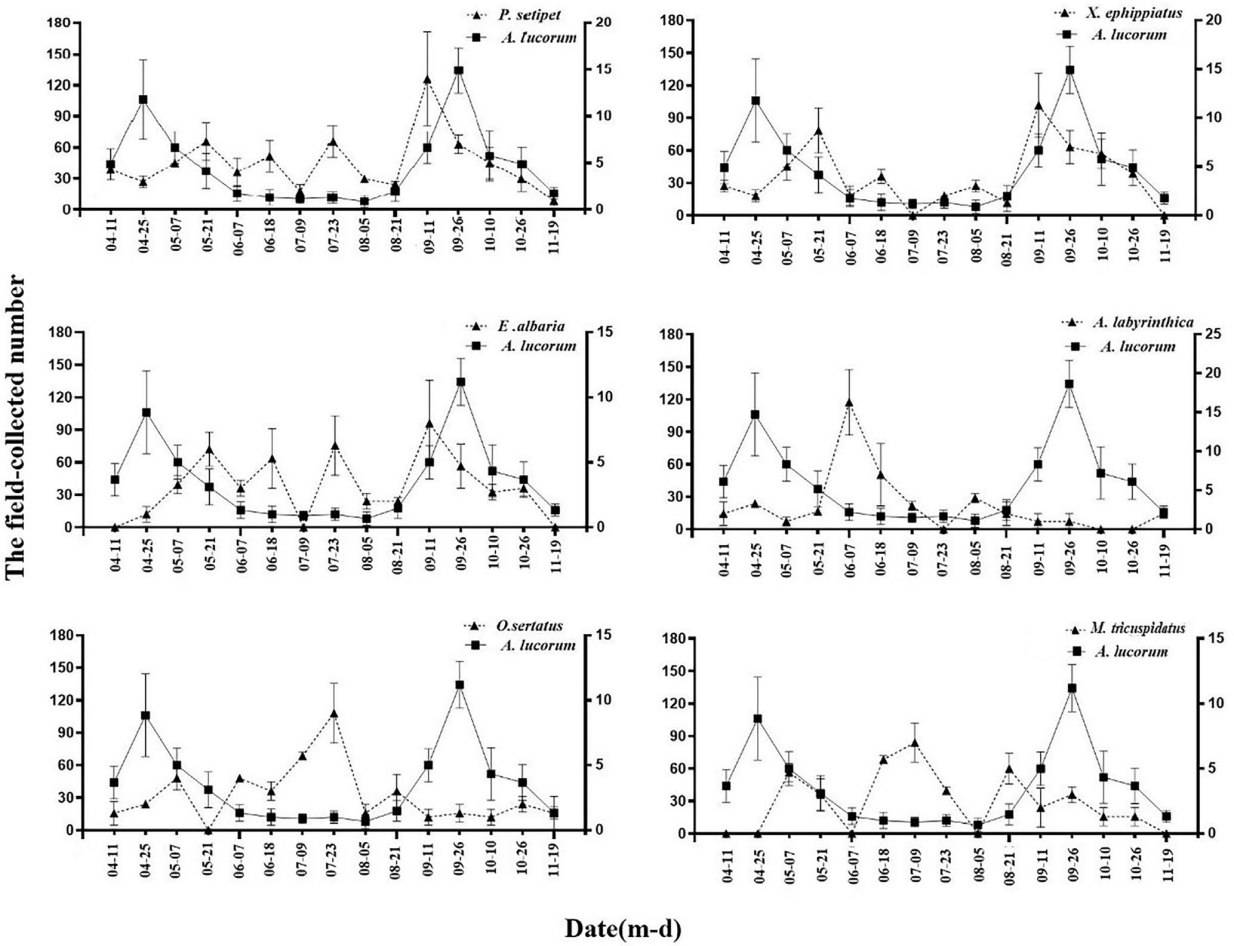
Population dynamics of *A. lucorum* and six dominant spider species in the canopies of tea. Values presented as mean ± SE (*N* = 3).

As shown in Figure 2, the population density of *A. lucorum* exhibited two distinct peaks, one in April and another in late September, which were significantly higher than the population density observed from June to August.

A total of 1174 spiders were collected and identified as belonging to 23 different species. Among these, *P. setipes* , *X. ephippiatus* , *A. labyrinthica* , *E. albaria* , *O. sertatus*, and *M. tricuspidatus* emerged as the most dominant species in the tea plantation. Their relative abundances were 19.2%, 15.3%, 11.5%, 12.1%, 10.2% and 9.3%, respectively. These six spider species were the focus of subsequent analyses. The population dynamics of the six spider species linked to *A. lucorum* are displayed in Figure 2, with their dominance and temporal niche overlap values detailed in Table 3. The six species of spiders, ranked from highest to lowest in the temporal niche overlap with *A. lucorum* , are as follows: *X. ephippiatus* , *P. setipes* , *E. albaria* , *M. tricuspidatus*, *O. sertatus* , and *A. labyrinthica* .

**TABLE 3:** The dominance of spider species and temporal niche overlap between spider species and *A. lucorum*.

Species	Dominance (%)	The temporal niche overlap value between spider species and *A. lucorum*
*P. setipet*	19.2	1.14
*X. ephippiatus*	15.3	1.29
*E. albaria*	12.1	1.08
*A. labyrinthica*	11.5	0.67
*O. sertatus*	10.2	0.74
*M. tricuspidatus*	9.3	0.86

### 3.4 Molecular Detection of Field Predation

The detection rate of *A. lucorum* DNA among these spider species was 4%. Specifically, 13.9% of *X. ephippiatus* , 7.3% of *M. tricuspidatus*, 2.2% of *P. setipes* , 1.4% of *E. albaria* , 0.7% of *A. labyrinthica* , and 2.3% of other spiders tested positive for the mirid bug DNA. The detection rates of *A. lucorum* DNA showed significant differences among these spider species (*χ* ^2^ = 60.9157, df = 6, *p* = 0.0001). Among these predators, *X. ephippiatus* and *M. tricuspidatus* showed significantly higher positive proportions compared to others (Table 4).

**TABLE 4:** Percentage of positive detection of *Apolygus lucorum* DNA from different spiders collected in tea plantation.

Species	Field collected number (juvenile and adult)	The positive *A. lucorum* DNA detection (%)	The detectability half‐life DS_50_ (h)	The weighted positive DNA detection (%)
*P. setipet*	225	2.2	33.35	1.94
*X. ephippiatus*	180	13.9	34.77	11.73
*E. albaria*	142	1.4	29.35	1.4
*A. labyrinthica*	135	0.7	36.18	0.57
*M. tricuspidatus*	109	7.3	47.95	4.46
*O. sertatus*	120	0	25.5	—

### 3.5 Predation Function Responses

**FIGURE 3:**
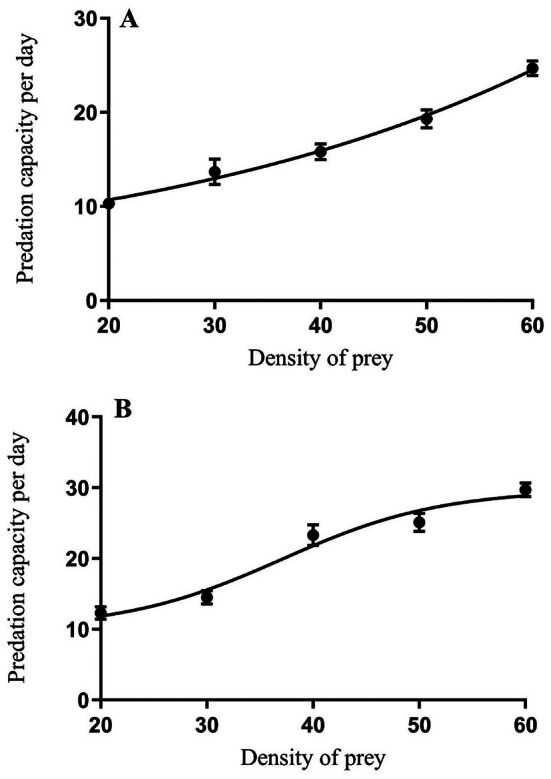
Daily average number of *A. lucorum* consumed by *X. ephippiatus* (A) and *M. tricuspidatus* (B).

The analysis showed that the predatory behavior of adult *X. ephippiatus* and *M. tricuspidatus* against *A. lucorum* aligned with the Holling Type II functional response model. The fitting results of the Holling Type II model showed that adult *X. ephippiatus* had a higher instantaneous attack rate on *A. lucorum* , reaching 0.61, and its theoretical maximum daily predation capacity was 108.3, which was higher than that of adult *M. tricuspidatus* (with a theoretical maximum daily predation capacity of 43.1 individuals) (Table 5, Figure 3).

**TABLE 5:** Parameters of functional response of spider predation on *Apolygus lucorum*.

Species	Functional response equation	*R* ^2^	a'	T_h_	N_a‐max_ (a'/Th)	*X* ^2^	*p*
*M. tricuspidatus*	Na = 0.55 × N/(1 + 0.007 × N)	0.81	0.55	0.01	43.1	1.61	0.987
*X. ephippiatus*	Na = 0.61 × N/(1 + 0.003 × N)	0.965	0.61	0.006	108.3	0.58	0.977

*Note:* Na‐max = a'/Th (the theoretical maximum number of prey consumed per day).

Predation by *X. ephippiatus* and *M. tricuspidatus* for different densities of *A. lucorum* displayed a similar trend (Figure 2). The quantity of predation of both spider species increased with prey density. However, under the same prey density, there were notable differences in the predation rates of *X. ephippiatus* and *M. tricuspidatus* on *A. lucorum*. At lower densities of *A. lucorum* , such as 20 and 30 individuals per dish, there was no significant difference in the predation rates between *X. ephippiatus* and *M. tricuspidatus* (20 individuals/per dish: *t* = 2.02, df = 18, *p* = 0.06; 30 individuals/per dish: *t* = 0.49, df = 18, *p* = 0.63). As the prey density increased, the quantity of predation of *X. ephippiatus* on *A. lucorum* rose rapidly. When the density of *A. lucorum* reached 40 individuals per dish, the quantity of predation of *X. ephippiatus* was significantly higher than that of *M. tricuspidatus* (*t* = 4.4152, df = 18, *p* = 0.0003).

## 4 Discussion

Natural enemies are critical biological factors in controlling pests. Understanding the population dynamics of pests and their natural enemies in the field, as well as elucidating the key periods of their occurrence and pest control, contributes to unraveling the succession patterns and ecological mechanisms of pest communities in crops. This study clarified the population dynamics of *A. lucorum* and spiders in tea plantations through field investigations. The mirid bug *A. lucorum* began to cause damage during the sprouting period of spring tea buds, with its population reaching its peak in late April. A large number of nymphs inhabited the tender buds, piercing‐sucking and causing harm. Subsequently, as the buds grew, the population density of mirid bugs progressively declined, maintaining a relatively low population density from June to August. By early September, as tea plants began to flower, the population density of *A. lucorum* had been gradually increasing, reaching its population peak again in late September. The emergence of natural enemy insects often exhibits a significant tracking relationship with their prey (Pan et al. 2020). Our field observations revealed that *P. setipes* was the predominant spider species inhabiting tea plant canopies, while the greatest temporal niche overlap was observed between *X. ephippiatus* and *A. lucorum* in the study region.

Since spiders represent one of the most abundant groups of natural predators in tea plantations, assessing their potential for pest control is of great importance. Previous research on predator roles has primarily centered on their dominance and the niche overlap between predators and their prey (Liu et al. 2002; Wang et al. 2008). However, empirical evidence regarding the extent and efficacy of predator‐pest interactions under natural field conditions remains limited. To address this gap, a comprehensive assessment of predation rates and the number of pests consumed by predators was conducted to better understand their role in pest control. The species‐specific primer technique, a widely utilized and effective method for gut content analysis, stands out as a highly efficient and economical approach for identifying prey remnants (Hosseini et al. 2012). Field‐collected predators are analyzed using designed species‐specific primers to enhance the comprehension of predator–prey interactions and minimize potential experimental biases. Here, we conducted molecular detection on the spider species collected from tea plantations and found that the rates of detecting the DNA of *A. lucorum* in the guts of *X. ephippiatus* and *M. tricuspidatus* were significantly higher than in other spiders, indicating that they are the primary predatory natural enemies of the green mirid bug. Although the population of *P. setipes* was higher than that of other spiders, its predation rate on the green mirid bug was significantly lower than that of *X. ephippiatus* and *M. tricuspidatus*; its role in controlling the green mirid bug was limited. In addition, the detection rates of *A. lucorum* DNA in *E. albaria* and *A. labyrinthica* were also very low, less than 2%. The detection rate of predators depends critically on the prey's detectable period, which is directly limited by the predator's digestive duration (Chen et al. 2000; King et al. 2008). Previous research suggests that extended periods of prey detectability offer greater advantages over shorter durations, primarily by enhancing the likelihood of capturing infrequent predation events (Agustí et al. 2003; Greenstone et al. 2014; Harper et al. 2005). Nevertheless, prolonged detectability periods can potentially lead to an overestimation of predation rates, as the inability to differentiate between recent and historical predation events may result in cumulative counting errors (Hagler and Naranjo 1997; Ma et al. 2005). Considering the potential differences in digestion rates among these spider species, we conducted laboratory experiments to measure their digestion times. The results showed no significant differences in digestion rates among the six spider species. To address these limitations, we implemented a weighted detection rate methodology, effectively accounting for variations in digestion times and enabling more accurate interpretation of field‐collected data. This suggests that, in the field, *E. albaria* and *A. labyrinthica* have limited effectiveness in controlling the green mirid bug.

To enhance data comprehension and interpretation from molecular analyses, and to improve the precision and dependability of the obtained data, we additionally performed a functional responses experiment. Understanding the functional responses of different predators to their prey, thoroughly exploring local natural enemy resources, and fully leveraging the potential of natural enemies in biological pest control are crucial for achieving green pest management strategies (Fantinou et al. 2012; Ganjisaffar and Perring 2015; Wyckhuys et al. 2013). Our results showed that the functional predatory responses of *X. ephippiatus* and *M. tricuspidatus* to *A. lucorum* fit the Type II Holling model. At the same density of *A. lucorum* , especially at higher prey densities such as 40 individuals per dish, the predation rate of adult *X. ephippiatus* on the green mirid bug was higher than that of *M. tricuspidatus*.

A systematic study was conducted at three levels—field population dynamics and assessed spiders' phenological overlap with *A. lucorum* , laboratory predation capacity evaluation, and prey detection rates within natural enemies—to investigate the control effects of spiders on *A. lucorum* in tea plantations. Our study clarified the occurrence patterns of the green mirid bug and its predatory spiders in tea gardens and elucidated the control effects of dominant predatory natural enemies, represented by *X. ephippiatus* , on the green mirid bug. The findings provide a scientific basis for further exploration and utilization of natural enemy resources, as well as enhancing green pest control strategies against the green mirid bug in tea plantations.

## Author Contributions

**Meng Zhang:** conceptualization (equal), investigation (equal), methodology (equal), resources (equal), writing – original draft (equal), writing – review and editing (equal). **Xiaodong Fu:** methodology (equal), resources (equal), writing – review and editing (equal). **Feiyang Li:** validation (equal), writing – review and editing (equal). **Yalan Zhu:** methodology (equal), writing – review and editing (equal). **Yu Gao:** methodology (equal), writing – review and editing (equal). **Dayu Zhang:** writing – review and editing (equal). **Qian Wang:** funding acquisition (equal), writing – original draft (equal), writing – review and editing (equal).

## Funding

This work was supported by the Zhejiang Provincial Natural Science Foundation of China under Grant No. LQ21C140001, National Natural Science Foundation of China (32102200).

## Conflicts of Interest

The authors declare no conflicts of interest.

## Data Availability

All data used in the study are included in this paper.
